# Brain tissue viscoelasticity in chronically shunted patients with headaches using Magnetic Resonance Elastography

**DOI:** 10.1186/2045-8118-12-S1-O30

**Published:** 2015-09-18

**Authors:** Kristy Tan, Adam L Sandler, Avital Meiri, Rick Abbott, James T Goodrich, Eric Barnhill, Mark E Wagshul

**Affiliations:** 1 Albert Einstein College of Medicine, USA; 2 Department of Neurological Surgery, Albert Einstein College of Medicine/Children's Hospital at Montefiore, Bronx NY, USA; 3 Clinical Research Imaging Centre, College of Medicine and Veterinary Medicine, University of Edinburgh, UK

## Introduction

Chronic headaches are a well-documented complaint of shunted hydrocephalic patients. However, it is also one of the signs of shunt malfunction. Cranial compliance deficiency may be a cause of chronic headaches in some chronically shunted patients with functioning shunts (often with slit or smaller than normal ventricles). This study aims to use a novel, non-invasive imaging technique, Magnetic Resonance Elastography (MRE) to investigate the role of brain viscoelasticity in pediatric hydrocephalic patients.

## Methods

Shunt-dependent patients who developed hydrocephalus as infants were selected. Preliminary results from 12 patients (age 15-37, median age 23) who suffer from chronic headaches (excluding patients with abnormally large ventricles, defined as ventricular volume < 25 cm3) are shown.

MRE was performed by inducing a mechanical wave at 30Hz, transmitted through the zygomatic arches. Wave propagation speed was used to estimate viscoelasticity (shear modulus, G*). Images were motion and distortion corrected using MRE magnitude and field maps respectively. Image segmentation was performed on registered high-resolution images to produce CSF, white and gray matter masks using FSL. Ventricular volume was calculated from the CSF component.

## Results

Good wave penetration was observed in MRE results allowing high quality data to be reconstructed. The viscoelasticity for gray matter was found to be positively correlated to white matter (R2=0.97, p<0.01). There was a positive correlation between the viscoelasticity of gray matter and ventricular volume that was statistically significant (R2=0.60, p=0.04). There was also a trend toward significance between the viscoelasticity of the white matter and ventricular volume (R2=0.49).

## Conclusions

This pilot study demonstrates that MRE could be a powerful diagnostic tool to be used in pediatric hydrocephalic patients. Preliminary results show the brain to be more viscoelastic as ventricle size increases in patients indicating that compliance could be key in understanding the cause of headaches in chronically shunted patients. This study is ongoing with the aim of comparing control and patient data and correlating viscoelasticity to headache severity.
